# Altered distribution of I_Ca_ impairs Ca release at the t-tubules of ventricular myocytes from failing hearts

**DOI:** 10.1016/j.yjmcc.2015.06.012

**Published:** 2015-09

**Authors:** Simon M. Bryant, Cherrie H.T. Kong, Judy Watson, Mark B. Cannell, Andrew F. James, Clive H. Orchard

**Affiliations:** School of Physiology and Pharmacology, Medical Sciences Building, University of Bristol, Bristol BS8 1TD, UK

**Keywords:** t-tubules, Ca imaging, Heart failure

## Abstract

In mammalian cardiac ventricular myocytes, Ca influx and release occur predominantly at t-tubules, ensuring synchronous Ca release throughout the cell. Heart failure is associated with disrupted t-tubule structure, but its effect on t-tubule function is less clear. We therefore investigated Ca influx and release at the t-tubules of ventricular myocytes isolated from rat hearts ~ 18 weeks after coronary artery ligation (CAL) or corresponding Sham operation. L-type Ca current (I_Ca_) was recorded using the whole-cell voltage-clamp technique in intact and detubulated myocytes; Ca release at t-tubules was monitored using confocal microscopy with voltage- and Ca-sensitive fluorophores. CAL was associated with cardiac and cellular hypertrophy, decreased ejection fraction, disruption of t-tubule structure and a smaller, slower Ca transient, but no change in ryanodine receptor distribution, L-type Ca channel expression, or I_Ca_ density. In Sham myocytes, I_Ca_ was located predominantly at the t-tubules, while in CAL myocytes, it was uniformly distributed between the t-tubule and surface membranes. Inhibition of protein kinase A with H-89 caused a greater decrease of t-tubular I_Ca_ in CAL than in Sham myocytes; in the presence of H-89, t-tubular I_Ca_ density was smaller in CAL than in Sham myocytes. The smaller t-tubular I_Ca_ in CAL myocytes was accompanied by increased latency and heterogeneity of SR Ca release at t-tubules, which could be mimicked by decreasing I_Ca_ using nifedipine. These data show that CAL decreases t-tubular I_Ca_ via a PKA-independent mechanism, thereby impairing Ca release at t-tubules and contributing to the altered excitation–contraction coupling observed in heart failure.

## Highlights

• Whole-cell Ca current (I_Ca_) density is not altered in myocytes from failing hearts.
• I_Ca_ density decreases in the t-tubules of myocytes from failing hearts.
• The decrease of t-tubular I_Ca_ is associated with impaired Ca release at t-tubules.
• These changes in Ca release can be mimicked by decreasing I_Ca_ using nifedipine.

## 1 Introduction

Normal contraction of the heart requires precise regulation of intracellular Ca release. This regulation is impaired in heart failure (HF), leading to a decrease in the speed and strength of contraction (see [1] for review), although the mechanisms remain unclear. Contraction is normally initiated by an action potential (AP), which is conducted into the interior of ventricular myocytes via transverse (t-) tubules, invaginations of the surface membrane that form a complex network within the cell [2,3]. The AP causes L-type Ca channels (LTCCs) to open, resulting in Ca influx (I_Ca_), which occurs mainly at t-tubules [4,5]. I_Ca_ activates ryanodine receptors (RyRs), which are located predominantly in sarcoplasmic reticulum (SR) membrane adjacent to t-tubules, triggering local Ca-induced Ca release, which undergoes spatial and temporal summation to produce the systolic Ca transient [6] that activates contraction. Thus coupling of Ca influx to Ca release, via local control of RyR activity, occurs mainly at t-tubules (for review, see [7]), ensuring near-synchronous Ca release and activation of contraction throughout the cell ([5,8]; for review, see [9]).

Impaired coupling between Ca influx and RyR activation was first demonstrated in an animal model of HF [10]. Subsequent studies have shown that HF is associated with reduced ability of SR to accumulate Ca and loss of t-tubules, but little or no change in whole-cell I_Ca_ [11]. These data suggested that the impaired coupling might be due simply to decreased SR Ca content and “orphaning” of RyRs due to local loss of t-tubules [12]. However, work showing an increase in LTCC current at the surface membrane in HF [13] suggests that other mechanisms may be modulating local LTCC activity and distribution of I_Ca_. Furthermore, failure of AP propagation and Ca release has recently been reported within some of the remaining t-tubule network in HF [14], suggesting local changes of ion channel function. A local reduction of I_Ca_ in the t-tubules could, potentially, decrease the probability of local Ca release and exacerbate the problems caused by the loss of t-tubules and reduced SR content in HF. We have, therefore, investigated whether I_Ca_ in the t-tubules is altered in an HF model and how this impacts on excitation-contraction coupling.

## 2 Methods

### 2.1 Surgical model of heart failure

Ligation of the left anterior descending coronary artery of adult male Wistar rats (CAL) results in heart failure ~ 16 weeks after surgery, with left ventricular hypertrophy and dilatation, and systolic and diastolic dysfunction [15]. Operations were performed under surgical anesthesia (ketamine 75 mg/kg, medetomidine 0.5 mg/kg, ip) with appropriate analgesia (buprenorphine 0.05 mg/kg, sc). The corresponding sham operation (Sham) was identical except that no tie was placed around the coronary artery. All procedures were performed in accordance with UK legislation and were approved by the University of Bristol Ethics Committee. Echocardiography was used to measure cardiac function in vivo, under isoflurane (1.5%) anesthesia using the Vevo 770 imaging system (Visualsonics Inc.) with an 11–24 MHz scanhead (RMV716). Images were taken in the long axis view and ejection fraction and left ventricular systolic and diastolic volumes calculated from 2 to 3 cardiac cycles.

### 2.2 Myocyte isolation and detubulation

Left ventricular myocytes were isolated from Sham and CAL animals 18.6 ± 0.3 or 18.5 ± 0.3 weeks after surgery, respectively. To isolate myocytes, animals were killed under pentobarbitone anesthesia, the heart quickly excised and myocytes isolated using our standard methods [16], and stored for 2–8 hours before use on the day of isolation; cells were isolated from the septum and left ventricular free wall, including the peri-infarct region, to investigate left ventricular changes in response to infarct and early stage HF. Myocyte detubulation (DT)—physical and functional uncoupling of the t-tubules from the surface membrane—was achieved using formamide-induced osmotic shock, as described previously [5,8]; data from intact and DT myocytes were obtained from different cells and are therefore unpaired.

### 2.3 Solutions

For patch-clamp experiments, cells were superfused with a solution that contained (in mmol/L): 133 NaCl, 1 MgSO_4_, 1 CaCl_2_, 1 Na_2_HPO_4_, 10 D-glucose, 10 HEPES, pH 7.4 (NaOH); 5 CsCl was added to inhibit K currents. The pipette solution contained (in mmol/L): 110 CsCl, 20 TEACl, 0.5 MgCl_2_, 5 MgATP, 5 BAPTA, 10 HEPES, 0.4 GTP-Tris, pH 7.2 (CsOH). The perfusion solution for Ca transient measurements was the same as that used for patch-clamping, except that CsCl was replaced with 5 mmol/L KCl. All experiments were performed at room temperature.

### 2.4 Recording and analysis of I_Ca_

Myocytes were placed in a chamber mounted on a Nikon Diaphot inverted microscope. Membrane currents and cell capacitance were recorded using the whole-cell patch-clamp technique, using an Axopatch 200B and Digidata 1322A A/D converter (Axon Instruments). pClamp 10 software (Axon Instruments) was used for data acquisition and analysis. Patch pipette resistance was typically 1.5–3 MΩ when filled with pipette solution. Pipette capacitance and series resistance were compensated by > 70%. I_Ca_ was elicited from a holding potential of − 80 mV, after a 100 ms step depolarization to − 40 mV (to inactivate I_Na_), by step depolarizations to voltages between − 50 and + 80 mV (10 mV steps) for 500 ms, before repolarization to the holding potential, at a frequency of 0.2 Hz.

I_Ca_ amplitude was measured as the difference between peak inward current and current at the end of the depolarizing pulse to give absolute current (pA), which was normalized to cell capacitance (a function of membrane area) to give current density (pA/pF). To correct for incomplete detubulation, the distribution of membrane capacitance and I_Ca_ between the t-tubule and surface membranes was calculated as follows:

$$\begin{array}{l} D_{TT}=\left(I_{N}–I_{D}\right)/\left(C_{N}–C_{D}\right)\\ C_{TT}=\left(C_{N}–C_{D}\right)/\left(1-\alpha \right)\\ I_{TT}=D_{TT}\;x\;C_{TT}\\ D_{SS}=\left(I_{D}–\alpha \;x\;I_{TT}\right)/\left(C_{D}–\alpha \;x\;C_{TT}\right)\\ C_{SS}=\left(C_{D}–\alpha \;x\;C_{TT}\right)\\ I_{SS}=D_{SS}\;x\;C_{SS} \end{array}$$

where C_N_ and C_D_ are membrane capacitance, and I_N_ and I_D_ are absolute I_Ca_ amplitude in intact and DT myocytes, respectively, and α is the proportion of t-tubule membrane remaining in DT myocytes, set to 0.16 (see below). C_SS_ and C_TT_, I_SS_ and I_TT_, and D_SS_ and D_TT_ are corrected values for membrane capacitance, I_Ca_ amplitude and I_Ca_ density, respectively, in the surface sarcolemmal (ss) and t-tubule (tt) membranes. Ca entry via LTCCs in the t-tubule membrane is calculated from the difference in I_Ca_ between intact and DT myocytes; Ca entry via LTCCs in the surface membrane is given by corrected I_Ca_ recorded from DT myocytes.

Inactivation of I_Ca_ was quantified by fitting the decay of the current between its peak and steady state to a double exponential function to give “fast” (τ_fast_) and “slow” (τ_slow_) time constants.

### 2.5 Confocal image acquisition and analysis

The cell membrane was stained by incubating cells with di-8-ANEPPS (5 μmol/L) for 15 minutes at room temperature. RyRs were labeled using standard immunocytochemical techniques [17]: in brief, cells were fixed in 4% paraformaldehyde in phosphate-buffered saline (PBS), permeabilized with 0.1% Triton-X100, and then incubated in 10% goat serum. After labeling with mouse anti-RyR2 antibody (1:200; MA3-916, Thermo Fisher Scientific, Massachusetts, USA) in PBS containing 5% goat serum and 2% bovine serum albumin, they were incubated with Alexa 647 goat anti-mouse antibody (1:200; A21235, Thermo Fisher) before being mounted in Vectashield with DAPI (Vector Laboratories, California, USA) on standard glass slides.

Images of stained cells were acquired using a confocal microscope (SP5-AOBS, Leica Microsystems, Wetzlar, Germany), with a 1.4 NA 63 × oil immersion objective and the pinhole set to 1 Airy unit. Di-8-ANEPPS was excited at 488 nm and fluorescence collected between 500 and 572 nm. RyR labeling was excited at 647 nm and fluorescence monitored between 650 and 750 nm; voxel size was ~ 120 nm in-plane and ~ 250 nm along the optical axis.

T-tubule staining was analyzed using custom routines written in IDL (version 6.3; Excelis Visual Information Solutions, Colorado, USA). Image stacks were deconvolved using the Richardson–Lucy algorithm and point-spread function (PSF) measured from sub-resolution fluorescent microspheres. Surface (sarcolemma) and internal (t-tubule) labeling were distinguished manually and the density of t-tubule staining was analyzed using two independent methods: (i) percentage of pixels retained in a binary image formed by thresholding above the mean background intensity, to give staining as a percentage of total area and (ii) a skeletonized image, in which t-tubules were reduced to single-pixel wide lines to avoid complications arising from possible changes in t-tubule width, to give length of t-tubule per unit area. Regularity of staining was determined from a two dimensional discrete Fast Fourier Transform (FFT) [23,25].

The regularity of RyR labelling was also assessed using FFT analysis. The 3D spatial density of RyR clusters was determined using a custom-written detection algorithm based on a matched filter detection strategy [18,19] and implemented in MATLAB (R2013a; Mathworks Incorporated, Massachusetts, USA). In brief, a 3D correlogram of the deconvolved image stack and microscope PSF was used to locate puncta by searching for the co-ordinates of the maximum correlation value. These locations were used to develop a 3D model of RyR cluster centers, which was refined until all labeling was accounted for. RyR cluster density was then expressed as number of clusters per μm^3^.

### 2.6 Western blot analysis

10 μg samples of myocyte lysates were run on 4–15% gradient SDS–PAGE gels and transferred onto Immobilon-P membrane. The blot was probed with anti-LTCC antibody (ACC-003; Alomone, Israel) or anti-GAPDH (G9545; Sigma) and protein bands visualized using relevant peroxidase-conjugated secondary antibodies, chemiluminescence, and autoradiography. Band density was measured using Image J (http://imagej.nih.gov/ij/) and normalized to GAPDH.

### 2.7 Confocal image acquisition and analysis of membrane potential and intracellular Ca

Simultaneous recording of local fluorescent voltage and Ca signals at a t-tubule was achieved by incubating isolated cells in 0.5–1 μg/mL di-4-AN(F)EPPTEA (31; a kind gift from Dr Leslie Loew) for 15 minutes and 5 μmol/L Fluo-4/AM (Life Technologies, CA, USA) for 25 minutes. Cells were then washed and imaged on a Leica SP5 confocal system in line-scan mode (0.71 ms/line, ~ 80 nm/pixel) to record both Ca- (516–560 nm) and voltage- (590–750 nm) sensitive signals, with excitation at 514 nm. Membrane staining was used to identify a t-tubule for voltage and Ca measurements by line-scanning. Cells were field-stimulated at 0.1 Hz via parallel Pt wires at 1.5 × threshold. Whole-cell Ca transients were obtained using line scans along the length of myocytes loaded with Fluo-4 as above, using either the Leica SP5 or a Zeiss Pascal confocal system.

Since di-4-AN(F)EPPTEA fluorescence decreases with membrane depolarization, a decrease in signal that was 2.5 standard deviations (SDs) below that of the pre-stimulus period was used to define the upstroke of the AP at the t-tubule. Latency of Ca release at each point along the scan was determined as the time between the upstroke of the AP and the time when the Ca signal became > 5 SD above the pre-stimulus value, multiplied by the maximum rate of rise in ΔF/F_0_/ms. The latter correction avoids the problem associated with a simple constant threshold, which would report an earlier onset for a signal with a faster rate of rise. The latency to time of maximum rate of rise of Ca was also determined, and the SD of latencies for each cell was used as a measure of the heterogeneity of release.

### 2.8 Statistics

Data are expressed as mean ± SEM. Paired and unpaired t-tests and 1- or 2-way ANOVA were used as appropriate with the Bonferroni *post hoc* test where applicable. The errors of derived variables, and the subsequent statistical analysis, were calculated using propagation of errors from the constituent measurements [20]. Statistical significance was taken as *p* < 0.05. n = number of observations; for electrophysiological data, n is given as c/h where c = the number of cells used from h hearts.

## 3 Results

### 3.1 Effects of coronary artery ligation (CAL) on cardiac and myocyte morphology

**Fig. 1:**
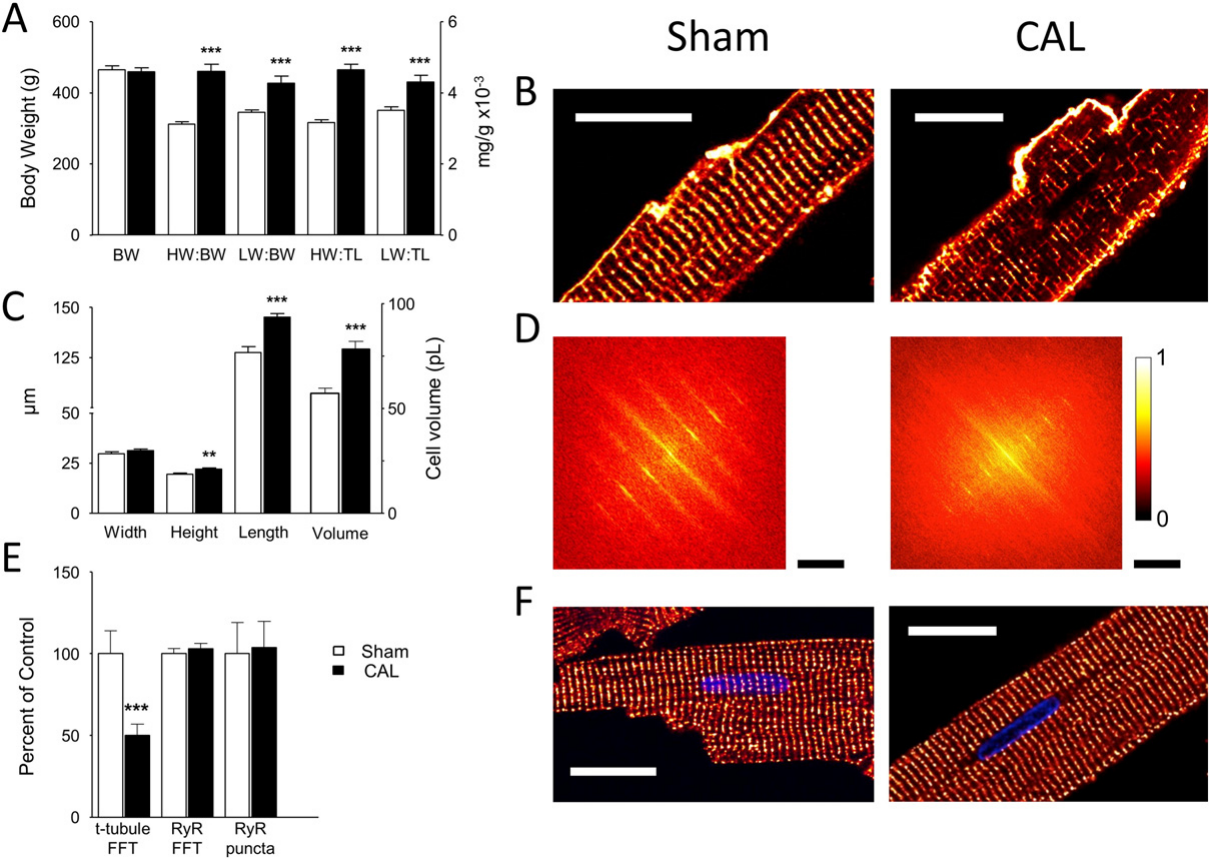
**Effects of CAL on cardiac and myocyte morphology.** A: Mean (± SEM) body weight (left axis), and heart weight (HW) and lung weight (LW) relative to body weight (BW) and tibia length (TL) in Sham (open bars, *n* = 21) and CAL (filled bars, *n* = 22) animals. B: Confocal images of mid-section slices from representative Sham (left) and CAL (right) myocytes stained with di-8-ANEPPS; scale bar 20 μm. C: Mean (± SEM) cell dimensions (left axis) and volume (right axis) of myocytes isolated from Sham (open bars, *n* = 48/4) and CAL (filled bars, *n* = 58/4) myocytes. D: 2D FFTs of the cells shown in B (cropped to half their original size to show the central portion comprising 0 to ~ 4^th^ harmonics). FFT values are normalized to the peak value and scaled (color bar on right). The scale bars show 0.94/μm for both Sham and CAL. E: Mean (± SEM) t-tubule power (Sham, *n* = 48; CAL, *n* = 58), RyR power and density of RyR clusters in Sham (open bars, *n* = 11) and CAL (filled bars, *n* = 10) myocytes, normalized to mean Sham data. **p < 0.01, ***p < 0.001 *vs* Sham. F: Representative confocal images of RyR labeling in single Sham (left) and CAL (right) myocytes, scale bar 20 μm.

Left ventricular myocytes were isolated from the hearts of animals that had undergone either Sham (n = 21) or CAL (n = 20) operations ~ 18 weeks previously. At the time of cell isolation, there was no significant difference in body weight (Fig. 1A) or tibia length (Sham: 45.1 ± 0.4 mm; CAL: 44.7 ± 0.4 mm) between the two groups, but CAL animals showed significant increases in heart and lung weights (HW and LW) relative to body weight and tibia length (BW and TL; Fig. 1A), decreased ejection fraction, from 76.6% to 38.7% (*p* < 0.01), and increased left ventricular diastolic and systolic volumes, from 182 to 408 μL and from 45 to 250 μL (*p* < 0.05), respectively, indicative of cardiac insufficiency and early stage heart failure.

Fig. 1B shows confocal images of representative Sham (left) and CAL (right) myocytes stained with di-8-ANEPPS. CAL caused an increase in cell size (Fig. 1C) and whole-cell membrane capacitance, which increased from 260 ± 9 (n = 37/13) to 365 ± 13 (n = 33/13) pF (p < 0.001). There was no significant change in t-tubule abundance: internal di-8-ANEPPS staining occupied 39 ± 4% of the cell area in Sham, and 42 ± 5% in CAL, myocytes; in skeletonized images, t-tubules occupied 0.58 ± 0.05 μm/μm^2^ in Sham (n = 10) and 0.52 ± 0.03 μm/μm^2^ in CAL (n = 22) myocytes. However, Sham myocytes showed a more regular transverse staining than CAL myocytes. Fig. 1D shows 2D FFT analysis of this staining in the cells shown in panel B. FFT analysis showed a decrease in power in myocytes isolated from CAL hearts (Fig. 1E) consistent with decreased t-tubule regularity. Fig. 1F shows representative confocal images of Sham and CAL myocytes stained for RyR, showing transverse bands of punctate labeling in both cell types. Fig. 1E shows mean data for 2D FFT and 3D puncta analysis of RyR staining, showing that there was no significant change in either the regularity of RyR distribution (Sham: 0.32 ± 0.02, n = 11; CAL: 0.33 ± 0.02, n = 10) or in the density of RyR clusters (Sham: 1.13 ± 0.15; CAL: 1.08 ± 0.16 puncta/μm^3^).

### 3.2 Effect of CAL on I_Ca_, LTCC expression and the Ca transient

**Fig. 2:**
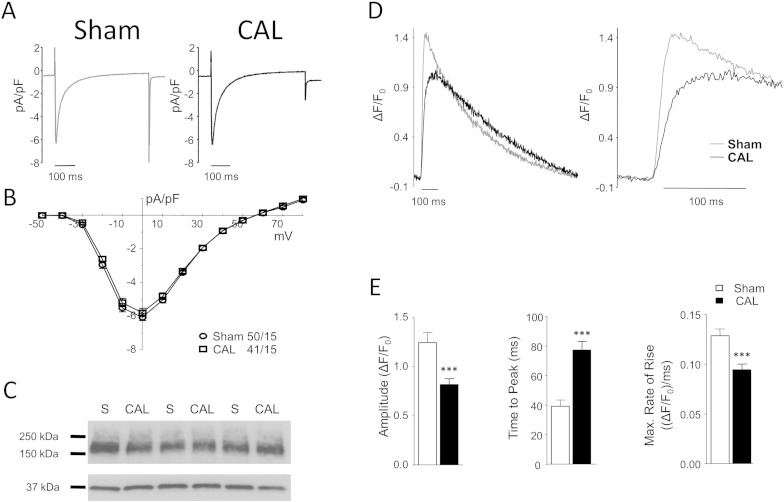
**Effect of CAL on I_Ca_, LTCC expression and the systolic Ca transient.** A: Representative L-type Ca currents (I_Ca_) recorded at 0 mV from Sham (left) and CAL (right) myocytes. B: Mean (± SEM) I_Ca_ density-voltage relations from Sham (circles, *n* = 50/15) and CAL (squares, *n* = 41/15) myocytes. C: Representative Western blots of Sham (S) and CAL lysates probed with antibodies against LTCC (top) or GAPDH (bottom). D: Representative whole-cell Ca transients from Sham (grey line) and CAL (black line) myocytes during field stimulation (left); the right panel shows the early rise on an expanded time scale. E: Mean (± SEM) peak ΔF/F_0_, time to peak (TTP), and rate of rise (ΔF/F_0_.ms^− 1^) of the Ca transient in Sham (open bars, *n* = 10) and CAL (filled bars, *n* = 16) myocytes. ^⁎⁎⁎^*p* < 0.001 *vs* Sham.

Fig. 2 shows representative recordings of I_Ca_ from Sham and CAL myocytes (Fig. 2A), the corresponding I_Ca_–voltage relationships (Fig. 2B), and Western blots of the alpha subunit of the LTCC (Ca_V_1.2; Fig. 2C). CAL had no significant effect on whole-cell I_Ca_ density, voltage dependence, or rate of inactivation (Table 1) or LTCC expression (Sham 100 ± 20.1, CAL 105 ± 14.5 arbitrary units, normalized to GAPDH, n = 3 in triplicate). Despite this, systolic Ca transients from CAL myocytes showed reduced peak amplitude, prolonged time to peak, and slower rate of rise than those from Sham cells (Fig. 2D and 2E), although the time taken for the transient to decay to 50% did not change significantly (Sham 211 ± 13 ms; CAL 213 ± 11 ms). The decreased rate of rise of the Ca transient might be expected as a result of the disrupted t-tubule structure [12,21,22]; however, the lack of effect on I_Ca_ was surprising, given the high density of I_Ca_ in the t-tubules [5].

### 3.3 Effect of CAL on I_Ca_ distribution between the t-tubular and surface membranes

We have previously shown that it is possible to dissect the relative densities of I_Ca_ in the t-tubule and surface membranes using osmotic detubulation (DT; [5,8]) which physically and functionally disconnects t-tubules from the surface membrane. The fractional decrease in cell capacitance following DT was not significantly different between the two groups (Sham, ~ 31.4%; CAL, ~ 25.8%; both *p* < 0.001 *vs* control). The efficacy of DT was confirmed by the decrease in internal membrane staining with di-8-ANEPPS, from 39 ± 4% to 6 ± 1% of cell area in Sham cells, and from 42 ± 5% to 7 ± 1% in CAL cells. Thus, the DT protocol disconnected ~ 84% of t-tubules in both cell types.

**Fig. 3:**
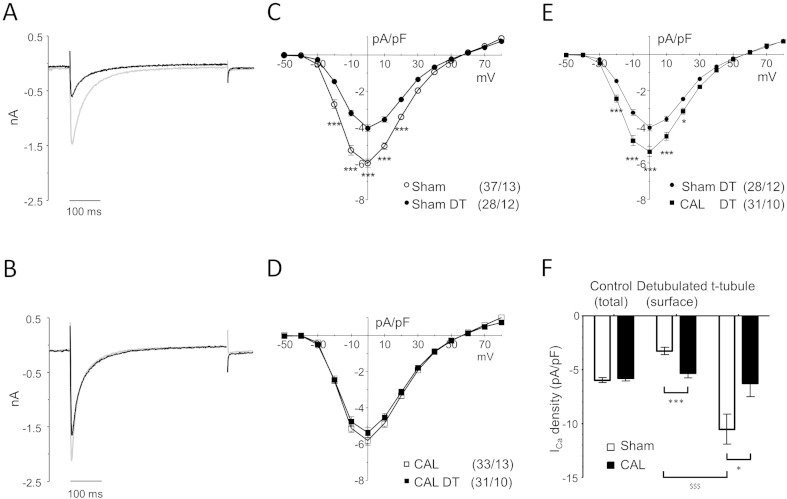
**Sub-cellular distribution of I_Ca_ in CAL myocytes**. A, B: Representative recordings of I_Ca_ obtained at 0 mV from intact (grey line) and detubulated (DT; black line) Sham (A) and CAL (B) myocytes. C: Mean (± SEM) I_Ca_ density-voltage relations in intact (open circles, *n* = 37/13) and detubulated (filled circles, *n* = 28/12) Sham myocytes. D: Mean (± SEM) I_Ca_ density–voltage relations in intact (open squares, *n* = 33/13) and detubulated (filled squares, *n* = 31/10) CAL myocytes. E: Mean (± SEM) I_Ca_ density–voltage relations in detubulated Sham (filled circles) and CAL (filled squares) myocytes. F: Mean (± SEM) I_Ca_ densities at 0 mV in intact Sham (n = 37) and CAL (n = 33) myocytes, and mean (± SEM) surface sarcolemmal and t-tubular I_Ca_ densities following correction for incomplete detubulation, as described in the text. ^⁎^*p* < 0.05, ^⁎⁎⁎^*p* < 0.001 CAL *vs* Sham; ^$$$^*p* < 0.001 surface *vs* t-tubule.

Fig. 3 shows representative records of absolute I_Ca_ recorded from intact and DT Sham (A) and CAL (B) myocytes, showing that DT resulted in loss of I_Ca_ in both groups of cells. In Sham myocytes, DT caused a greater decrease of I_Ca_ (55.2% at 0 mV) than cell capacitance (31.4%), resulting in a 32.6% decrease in I_Ca_ density (*p* < 0.001; Table 1 and Fig. 3C). The lower I_Ca_ density in DT cells shows that I_Ca_ is lower in the surface membrane than in the t-tubules. However, in CAL myocytes, DT caused a similar decrease of I_Ca_ (28.7% at 0 mV) and cell capacitance (25.8%), and thus little change in I_Ca_ density (− 7.5%; ns; Table 1 and Fig. 3D); this shows that I_Ca_ is more uniformly distributed between the t-tubule and surface membranes in these cells. However, I_Ca_ recorded from DT myocytes will include I_Ca_ from the remaining ~ 16% of t-tubules (above). We therefore corrected I_Ca_ recorded from DT myocytes for I_Ca_ in the remaining t-tubules, assuming that they have the same current density as those disconnected by DT in each group (see Methods). Fig. 3F shows I_Ca_ density in the surface and t-tubule membranes after correction: in Sham myocytes, I_Ca_ density in the t-tubules was significantly larger (× 3.2) than at the cell surface (− 10.53 *vs* − 3.28 pA/pF), while in CAL myocytes, I_Ca_ density was not significantly different (× 1.2) in the t-tubule and surface membranes (− 6.28 *vs* − 5.34 pA/pF). The corresponding ratios before correction were × 2.7 and × 1.2, respectively. In CAL myocytes, t-tubular I_Ca_ density was significantly lower, and surface membrane I_Ca_ density significantly higher, than in Sham. Thus in Sham myocytes, I_Ca_ density in the t-tubules is higher than in the surface membrane, as reported previously [5]; however, in CAL myocytes, I_Ca_ is uniformly distributed between the t-tubule and surface membranes due to a decrease in I_Ca_ density in the t-tubules and an increase at the cell surface.

Inactivation of I_Ca_ was prolonged in Sham and CAL myocytes after DT (Table 1), consistent with more rapid inactivation of the t-tubular component of I_Ca_, as reported previously [23], in both cell types. Thus, the Ca-dependent inactivation that underlies the more rapid inactivation of t-tubular I_Ca_ [24] appears to persist in CAL myocytes, despite loss of t-tubular I_Ca_, suggesting different underlying mechanisms.

Previous work has shown that I_Ca_ distribution is modulated by differences in basal protein kinase A (PKA)-dependent phosphorylation of LTCCs at the t-tubule and surface membranes [4,13], so that the decrease in t-tubular I_Ca_ in CAL myocytes might be explained by reduced LTCC phosphorylation at the t-tubules. To test this idea, we used the PKA inhibitor H-89 (20 μM) to decrease LTCC phosphorylation [25,26], since reduced basal phosphorylation of t-tubular LTCCs would be expected to result in H-89 causing a smaller decrease in phosphorylation (and thus t-tubular I_Ca_) in CAL than in Sham cells. H-89 decreased t-tubular I_Ca_ density, determined as described above, in both groups of cells, by 44% in Sham and 76% in CAL myocytes, so that in the presence of H-89, I_Ca_ density in the t-tubules was smaller in CAL than in Sham myocytes (Sham, 7.24 ± 1.2 *vs* CAL, 1.74 ± 0.6 pA/pF, *p* < 0.001). The larger fractional decrease of t-tubular I_Ca_ caused by H-89 and the lower t-tubular I_Ca_ density in the presence of H-89 in CAL myocytes suggest higher basal LTCC phosphorylation levels and lower density of LTCC in the t-tubules of CAL myocytes compared to Sham. However, the increased basal phosphorylation of t-tubular LTCC in CAL myocytes neither explains nor compensates for the decrease of t-tubular LTCC density.

### 3.4 Effect of CAL on local SR Ca release at the t-tubules

To investigate whether the decrease of I_Ca_ density in the t-tubules of CAL myocytes (Fig. 3) has functional consequences for Ca release at the t-tubules during normal excitation–contraction coupling (i.e. during action potentials), membrane potential and intracellular Ca signals were recorded simultaneously along t-tubules in field-stimulated myocytes. Cells were scanned transversely along a t-tubule identified by the voltage-sensitive dye di-4-AN(F)EPPTEA while Ca was monitored using Fluo-4. The voltage signal was used to detect the upstroke of the AP and hence the delay to the start of local Ca release and also ensured that observed changes were due to changes in t-tubule function rather than local action potential failure [14].

**Fig. 4:**
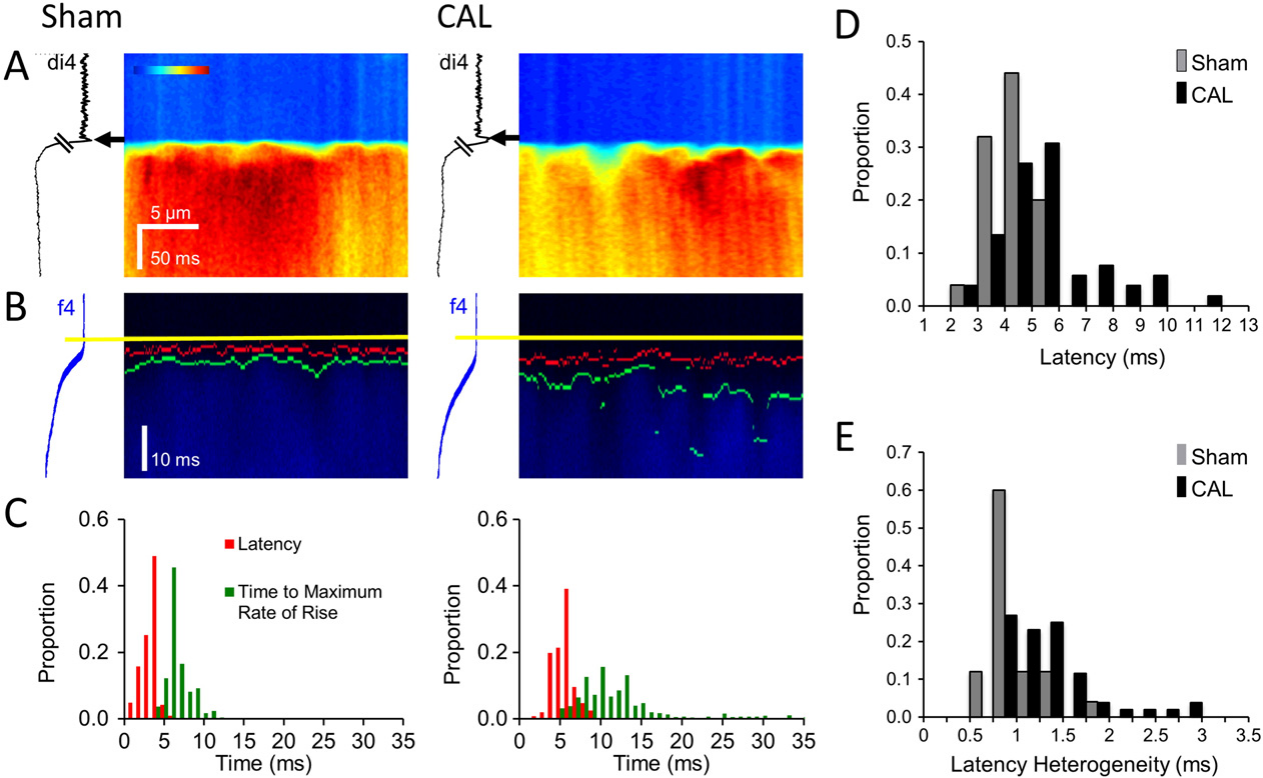
**Effect of CAL on Ca release at t-tubules**. A: Main panels show transverse line-scan images of Ca transients (field-stimulated at 0.1 Hz) recorded at a t-tubule in representative Sham (left) and CAL (right) myocytes; the traces to the left of each panel show average di-4-AN(F)EPPTEA (di4) fluorescence against time; arrows indicate the time of the t-tubule action potential. B: Traces shown in A on an expanded time scale. The start of the action potential is indicated by yellow lines, the start of the Ca transient by red lines, and the time of the maximum rate of rise of the Ca transient by green lines (see Methods); the traces to the left of each panel show average fluo-4 (f4) fluorescence against time. C: Histograms of latency (red) and time to maximum rate of rise (green) from the cells shown in A and B. D: Mean data for latency of Ca transient; Sham (grey bars; *n* = 25/4) had a mean latency of 3.3 ms, CAL (black bars; *n* = 54/6) had a mean latency of 5.5 ms; 4–7 Ca transients recorded from each myocyte. Bin size 1 ms. E: Mean data for heterogeneity of latency; Sham (grey bars; *n* = 25/4) and CAL (black bars; *n* = 54/6). Bin size 0.2 ms. See Table 2 for mean data.

Fig. 4A shows representative line-scan images obtained along single t-tubules in Sham and CAL myocytes (time running from top to bottom). Fig. 4B shows the same data at a faster time scale, showing the measured times of the AP upstroke at the t-tubule (yellow lines), the start of local Ca release (red lines), and the time of the maximum rate of rise of Ca release (green lines) along the t-tubule. Quantification of these latencies is shown in Fig. 4C, which shows the measured delays from the upstroke of the AP in the t-tubule membrane to the start of Ca release (red bars) and to the maximum rate of rise of Ca (green bars). It is clear that the delay for Ca release at the t-tubule was both longer and more variable in CAL myocytes. Pooled data show that CAL was associated with significantly prolonged latencies to the start (Fig. 4D; Table 2) and to the maximum rate of rise of Ca (Table 2), as well as increased heterogeneity in the time to the start, and to the maximum rate of rise, of Ca release (Fig. 4E; Table 2).

**Fig. 5:**
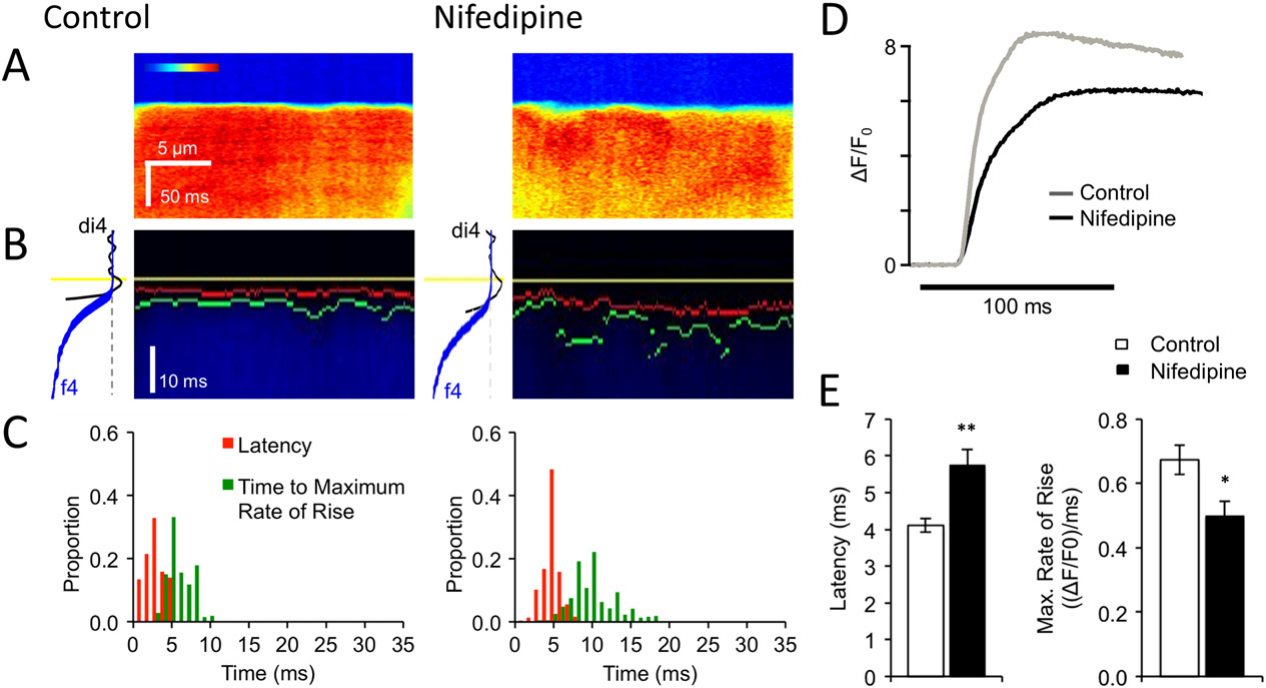
**Effect of nifedipine on Ca release**. A: Main panels show transverse line-scan images of Ca transients (field stimulated at 0.1 Hz) recorded at a t-tubule in representative myocytes in the absence (left) and presence (right) of nifedipine. B: Traces shown in A on an expanded time scale. The start of the action potential is indicated by yellow lines, the start of the Ca transient by red lines, and the time of the maximum rate of rise of the Ca transient by green lines (see Methods); the traces to the left of each panel show average di4 (black) and f4 (blue) fluorescence against time. C: Histograms of latency (red) and time to maximum rate of rise (green) from the cell shown in A and B. See Table 2 for mean data. D. Representative whole-cell Ca transients in the absence (grey line) and presence (black line) of nifedipine during field stimulation. E. Mean (± SEM) latency and rate of rise (ΔF/F_0_/ms) of the whole-cell Ca transient in the absence (open bars, *n* = 12) and presence (filled bars, *n* = 12) of nifedipine. ^⁎⁎⁎^*p* < 0.001 *vs* Control.

To test whether these changes could be explained by the decrease of I_Ca_ density in the t-tubules of CAL myocytes, we examined the effect of decreasing I_Ca_ using the LTCC blocker nifedipine (2 × 10^− 7^ mol/L) which decreased I_Ca_ by 51 ± 4%, n = 3, which is similar to previous studies [27] and comparable to the ~ 41% decrease in t-tubular I_Ca_ observed in CAL myocytes (Fig. 3 F). Fig. 5A–C illustrate that reducing I_Ca,_ with nifedipine had similar effects to CAL on local Ca release at a t-tubule: the increase in latency and heterogeneity caused by nifedipine was not significantly different from that caused by CAL (Table 2), showing that local reduction in I_Ca_ in the t-tubules can explain the changes of local Ca release observed in CAL myocytes. Nifedipine also recapitulated the effect of CAL on the rate of rise of the whole-cell Ca transient (*cf* Fig. 2). Fig. 5D–E show that nifedipine slowed the maximum rate of rise of the Ca transient by a similar extent (26.0%; control: 0.67 ± 0.05 ΔF/F_0_/ms, n = 12; nifedipine: 0.50 ± 0.05 ΔF/F_0_/ms; n = 12; *p* < 0.05) to that observed in CAL myocytes (26.3%, Fig. 2D), consistent with the idea that decreased t-tubular I_Ca_ contributes significantly to the slowed systolic Ca transient observed in CAL myocytes.

## 4 Discussion

Previous studies have shown increased spatiotemporal heterogeneity of Ca release due to loss of t-tubules, or due to t-tubules that do not generate an AP [14,21,22,28]. The present work shows for the first time that HF also results in redistribution of I_Ca_ away from the t-tubules, and that this decrease in t-tubular I_Ca_ impairs Ca release even at t-tubules that are generating an AP.

### 4.1 Altered distribution of I_Ca_ following CAL

The present study shows that whole-cell I_Ca_ density is not altered during the cellular hypertrophy associated with early stage HF, in agreement with previous work [11,13,29]. However, this lack of change results from differential effects at the cell surface and t-tubules, with I_Ca_ density decreasing at the t-tubules and increasing at the cell surface, so that it is more uniformly distributed in CAL myocytes. These changes are not due simply to the increased membrane area associated with hypertrophy, because as well as I_Ca_ density, absolute I_Ca_ also decreased (by 31%) in the t-tubules of CAL myocytes, and because in the absence of other changes the increase in surface area would decrease I_Ca_ density at the cell surface. Nor can the reduced t-tubular I_Ca_ in CAL myocytes be explained by decreased LTCC phosphorylation, because application of H-89 showed increased PKA-dependent stimulation of t-tubular LTCCs in these cells. The decrease in t-tubular LTCC availability could be explained by a decrease in the number of functional LTCCs, which could be due to decreased trafficking and insertion of LTCCs in t-tubule membrane, as we detected no change in LTCC expression by Western blotting, or to an increase in the number of non-functional LTCCs. The loss of functional LTCCs is partially offset by the increase in t-tubular LTCC phosphorylation, which could help explain why whole-cell I_Ca_ density is unchanged in CAL myocytes despite cellular hypertrophy and unaltered LTCC expression [13] and is accompanied by an increase at the surface membrane in these cells. To explore these ideas further using immunostaining would be problematic because (i) a given optical volume at the cell surface will contain a greater membrane area than the same volume (containing t-tubules) within the cell and (ii) the resolution of confocal microscopy means that surface label sampling will include fluorescence from t-tubule labeling close to the surface (which our data suggest will be different in CAL and Sham myocytes) while in the cell interior it will sample only t-tubule labeling. These problems will be exacerbated by the differences in t-tubule structure between Sham and CAL myocytes. (iii) It is not possible to distinguish between protein trafficking to the membrane and protein inserted and functional in the membrane. Thus, staining intensity measured from a region of cell does not solely depend on membrane protein density, which in turn does not necessarily reflect function, which depends on the presence of functional channels within the membrane and their activity, which can be altered by local changes in phosphorylation (present study; [4]). It is also difficult to quantify differences between Sham and CAL myocytes due to lack of a reference point, making correlation of staining and electrophysiology problematic. In contrast, measurement of I_Ca_ density enables the physiologically relevant distribution of protein function to be determined, even though it may not reflect local protein expression. It is, however, worth noting that RyR staining was used to determine the pattern of staining rather than to provide a quantitative comparison of staining intensity between different regions of the cell or between groups, thus circumventing these problems.

Nevertheless, in failing human ventricular myocytes, decreased t-tubular LTCC expression has been reported [30], and decreased t-tubular I_Ca_ has also been inferred from measurements of whole-cell I_Ca_ and single channel Ca currents at the cell surface [13], although the functional consequences of these changes were not demonstrated. A decrease in t-tubular I_Ca_ in HF has also previously been demonstrated following chronic treatment of mice with isoprenaline [31], although the relative effects of chronic PKA activation and HF were unclear, since PKA-induced phosphorylation has been implicated in the t-tubular localisation of I_Ca_ [4].

Since our data suggest changes in LTCC location in HF, a key question is what causes this redistribution. The mechanism(s) by which LTCCs are normally targeted predominantly to the t-tubules is incompletely understood [32], although BIN-1 may play an important role [30]. The mechanisms that alter the selective targeting to different surface membrane compartments in early HF should form the basis of future studies since it clearly has important implications for the uniformity of Ca release. The redistribution is, however, reminiscent of the redistribution of β_2_-adrenoceptors from the t-tubules in normal myocytes to a more uniform distribution in CAL myocytes [33,34], as a result of loss of Caveolin-3 dependent localization [16,35]. The t-tubule disruption and more uniform distribution of I_Ca_ in CAL myocytes is also suggestive of the situation in neonatal myocytes in which I_Ca_ is carried predominantly by surface membrane LTCCs, and the t-tubule network is less developed than in the adult myocyte (e.g. [36]). A shift in protein expression pattern toward more juvenile forms has been noted previously in HF [37] so that the observed changes in I_Ca_ distribution may reflect cellular reprogramming toward a neonatal phenotype.

### 4.2 Consequences of altered distribution of I_Ca_ in CAL myocytes

The distribution of RyRs appeared essentially unchanged in HF despite disruption of t-tubule structure. Although loss of t-tubules in HF can decrease Ca transient uniformity by “orphaning” RyRs [12], this complication was avoided in the present study by recording from regions of the cell adjacent to t-tubules. The decreased I_Ca_ density in the t-tubules of CAL myocytes resulted in increased spatiotemporal inhomogeneity of local Ca release; this novel mechanism will contribute to the decreased synchrony of Ca release in HF [10], even in regions of the cell that have not suffered loss of t-tubules or action potential failure [14]. Any compensatory increase in I_Ca_ density at the cell surface is unlikely to offset the detrimental effects of decreased Ca release at the t-tubules since it is not accompanied by redistribution of RyRs, and in any case, Ca release at the cell surface does not produce synchronous Ca release and contraction [5].

The increased inhomogeneity of Ca release at existing t-tubules is unlikely to be due to changes in the time course of I_Ca_, given its unaltered rate of inactivation, or to desynchronous depolarization along the t-tubule, given the space constant of cardiac myocytes. Although action potential prolongation has been reported in HF [1], this cannot account for the decreased uniformity of Ca release, which was measured during the first few ms after the upstroke of the action potential. However, previous work has shown that reducing I_Ca_ (under voltage clamp) reduces Ca uniformity by decreasing Ca spark probability [38], and we have shown that reducing I_Ca_ in control myocytes using nifedipine (which would shorten the action potential) recapitulates the effect of CAL on the latency and heterogeneity of Ca release at the t-tubules. This shows that the reduction in local I_Ca_ density in CAL myocytes can explain the observed decrease in Ca transient uniformity, although this does not exclude other possible changes within the cell playing additional role(s). This effect of LTCC redistribution is less marked than that due to physical loss of t-tubules [21,22] but is, nevertheless, an important contributor to the reduced rate of rise of the Ca transient.

Despite the change in the rising phase of the Ca transient, the time course of decay did not change significantly, in agreement with some, but not all, previous studies [1]; it has been suggested that an increase in Na/Ca exchange function may compensate for a moderate decrease in SR Ca-ATPase function in early HF [39,40], which may account for this observation.

### 4.3 Changes in t-tubule structure in CAL myocytes

In addition to changes in t-tubule function, we observed disruption of t-tubule structure in CAL myocytes, in agreement with previous work [21,22,41,42]. However, optical measurements of t-tubule staining and loss of cell capacitance following DT both showed no significant difference between Sham and CAL myocytes, demonstrating that there was no difference between Sham and CAL myocytes in the fraction of the cell membrane within the t-tubules. This was unexpected, given the disruption of t-tubule structure. However, dilation of t-tubules has been reported during progression to HF [43,44], which would tend to increase capacitance and staining per unit length of t-tubule, so that these measurements may reflect shorter, more dilated, t-tubules in CAL myocytes. In addition, these measurements represent area of membrane, and a similar fraction of membrane area in the larger CAL myocytes implies a smaller area of t-tubule membrane (a square function) per unit volume of cell (a cubic function). The disruption in t-tubule structure was not accompanied by marked changes in RyR distribution, suggesting that RyR distribution and t-tubule structure are maintained independently [12], although in more advanced (human) HF, a small decrease in RyR cluster density, accompanied by greater disruption of t-tubule organization, has been reported [44].

## 5 Conclusions

The present work shows altered t-tubule function following CAL, with a decrease in I_Ca_ density at the t-tubules, and an increase at the surface membrane. This results in increased variability and delay of Ca release even where t-tubules are preserved, which contributes to the slowed rate of rise of the systolic Ca transient. Since overall I_Ca_ density is not changed, HF must selectively alter protein trafficking and/or local activity, although why the t-tubule and surface membranes act as different compartments in this regard is unknown.

## Funding

This work was supported by the British Heart Foundation (grants PG/10/91/28644 and RG/12/10/29802).

## Disclosures

None.

## Figures and Tables

**Table 1:** Membrane capacitance and characteristics of I_Ca_ measured at 0 mV in intact (A) and detubulated (B) Sham and CAL myocytes (before correction for incomplete detubulation).

	Sham	CAL
*A: I_Ca_ properties recorded in intact myocytes*		
n (cells)	37	33
Capacity (pF)	260 ± 9	365 ± 13⁎⁎⁎
Peak I_Ca_ (pA)	− 1561 ± 81	− 2056 ± 74⁎⁎⁎
Peak I_Ca_ (pA/pF)	− 6.0 ± 0.2	− 5.8 ± 0.3
I_Ca_ inactivation		
Tau_fast_	23.9 ± 1.0	23.0 ± 1.1
Tau_slow_	97.2 ± 1.9	93.9 ± 2.4

*B: I_Ca_ properties recorded in detubulated myocytes*		
n (cells)	28	31
Capacity (pF)	178 ± 9 †††	271 ± 10 ⁎⁎⁎,†††
Peak I_Ca_ (pA)	− 699 ± 31 †††	− 1465 ± 98 ⁎⁎⁎,†††
Peak I_Ca_ (pA/pF)	− 4.0 ± 0.2 †††	− 5.4 ± 0.3⁎⁎⁎
I_Ca_ inactivation		
Tau_fast_	26.3 ± 1.1	23.2 ± 1.0⁎
Tau_slow_	110.0 ± 3.4 †††	103.6 ± 4.0 †

^⁎⁎^*p* < 0.01. vs Sham.

†† *p* < 0.01 vs control (intact) myocytes.

⁎ *p* < 0.05 *vs* Sham.

⁎⁎⁎ *p* < 0.001 *vs* Sham.

† *p* < 0.05 *vs* control (intact) myocytes.

††† *p* < 0.001 *vs* control (intact) myocytes.

**Table 2:** The effect of CAL and nifedipine on the latency and heterogeneity of local Ca release at the t-tubule.

Intervention	Surgery		Reduce I_Ca_	
Sham	CAL	Control	Nifedipine
n (cells)	25	52	12	11
Latency to initial rise of Ca (ms)	3.29 ± 0.16	5.51 ± 0.26⁎⁎⁎	3.73 ± 0.16	5.37 ± 0.49⁎⁎
Heterogeneity of initial rise of Ca (ms)	0.99 ± 0.05	1.36 ± 0.07⁎⁎	0.95 ± 0.09	1.29 ± 0.11⁎
Latency to max. rate of rise of Ca (ms)	7.71 ± 0.29	11.55 ± 0.50⁎⁎⁎	7.47 ± 0.35	10.44 ± 1.02⁎
Heterogeneity of max. rate of rise of Ca (ms)	1.99 ± 0.15	3.51 ± 0.23⁎⁎⁎	1.64 ± 0.17	3.11 ± 0.49⁎

All values are in ms. The control values for each variable were not significantly different between the groups, nor were any of the values significantly different between CAL and nifedipine.

⁎ *p* < 0.05.

⁎⁎ *p* < 0.01.

⁎⁎⁎ *p* < 0.001 *vs* relevant control.
